# An OX-Tra’Ordinary Tale: The Role of OX40 and OX40L in Atopic Dermatitis

**DOI:** 10.3390/cells13070587

**Published:** 2024-03-28

**Authors:** Kaviyon Sadrolashrafi, Lily Guo, Robin Kikuchi, Audrey Hao, Rebecca K. Yamamoto, Hannah C. Tolson, Sara N. Bilimoria, Danielle K. Yee, April W. Armstrong

**Affiliations:** Division of Dermatology, Department of Medicine, David Geffen School of Medicine at UCLA, Los Angeles, CA 90095, USA

**Keywords:** OX40, OX40L, atopic dermatitis, AD, eczema, cytokines, inflammation, AD pathogenesis, T cells, monoclonal antibodies

## Abstract

The transmembrane glycoprotein OX40 receptor (OX40) and its ligand, OX40L, are instrumental modulators of the adaptive immune response in humans. OX40 functions as a costimulatory molecule that promotes T cell activation, differentiation, and survival through ligation with OX40L. T cells play an integral role in the pathogenesis of several inflammatory skin conditions, including atopic dermatitis (AD). In particular, T helper 2 (T_H_2) cells strongly contribute to AD pathogenesis via the production of cytokines associated with type 2 inflammation (e.g., IL-4, IL-5, IL-13, and IL-31) that lead to skin barrier dysfunction and pruritus. The OX40-OX40L interaction also promotes the activation and proliferation of other T helper cell populations (e.g., T_H_1, T_H_22, and T_H_17), and AD patients have demonstrated higher levels of OX40 expression on peripheral blood mononuclear cells than healthy controls. As such, the OX40-OX40L pathway is a potential target for AD treatment. Novel therapies targeting the OX40 pathway are currently in development, several of which have demonstrated promising safety and efficacy results in patients with moderate-to-severe AD. Herein, we review the function of OX40 and the OX40-OX40L signaling pathway, their role in AD pathogenesis, and emerging therapies targeting OX40-OX40L that may offer insights into the future of AD management.

## 1. Introduction

Atopic dermatitis (AD) is a chronic inflammatory disease that affects up to 25% of children and 7–10% of adults [1]. AD typically presents in early childhood with acute flares of intensely pruritic and eczematous skin lesions [2]. These symptoms are potentially debilitating and may negatively impact many aspects of a patient’s quality of life, including sleep quality, self-esteem, school and work productivity, and mental health outcomes [1,2]. Some AD patients may develop other atopic conditions like asthma, allergic rhinitis, rhinoconjunctivitis, and food allergy [2,3,4]. AD may also be associated with a myriad of non-atopic comorbidities that are potentially related to systemic organ dysfunction caused by chronic inflammation, such as cardiovascular complications and metabolic disorders [3,5]. These associations emphasize the significant burden of AD on affected individuals, especially those with severe disease.

AD pathogenesis principally involves T cell-mediated processes that generate acute and chronic inflammatory responses [6,7]. Many treatment options aim to decrease inflammation through the modulation of the T cell response. Topical immunosuppressants like glucocorticoids, calcineurin inhibitors (e.g., tacrolimus and pimecrolimus), and phosphodiesterase-4 inhibitors (e.g., crisaborole) are generally recommended as first-line treatments for mild-to-moderate AD [4] as they help target local inflammation [1,3]. The landscape of available treatments for moderate-to-severe AD has rapidly advanced in recent years due to the identification of key immunologic pathways that have been shown to drive systemic inflammation [3,8,9]. Therapies that target these pathways include short-term phototherapy, systemic immunosuppressants (e.g., cyclosporine, azathioprine, methotrexate, and oral corticosteroids), Janus kinase (JAK) inhibitors (e.g., upadacitinib and abrocitinib), and monoclonal antibodies that block specific cytokines (i.e., biologics) [1,3,8,10].

While targeted biologic therapies like dupilumab (an IL-4Rα inhibitor) and tralokinumab (an IL-13 inhibitor) have substantially decreased the disease burden for many patients with moderate-to-severe AD, some still struggle to achieve a desired level of disease control [1,9,11]. As such, a therapeutic need exists for patients with moderate-to-severe AD who do not adequately respond to available treatments. AD pathophysiology is complex and involves many interconnected immunologic pathways. Therefore, identifying additional immune pathways that may contribute to AD pathogenesis can potentially lead to the development of novel therapies that allow a broader range of AD patients to optimize their long-term disease control.

The transmembrane glycoprotein OX40 receptor (OX40/CD134/TNFRSF4) and its ligand OX40L (CD252/TNFSF4) influence the production of different T cell populations and may be important for AD pathogenesis [12,13,14]. OX40 and OX40L are members of the tumor necrosis factor receptor (TNFR)/tumor necrosis factor (TNF) superfamily [12,15,16]. TNFR/TNF superfamily members are immune checkpoint molecules that can augment the T cell response through costimulation [15,16,17,18,19]. The interaction between OX40 and OX40L promotes T cell activation, differentiation, and survival through various mechanisms [12,15,20,21]. The inhibition of this interaction can potentially attenuate T cell-mediated processes that contribute to AD pathogenesis [15,22,23]. As such, OX40 and OX40L may serve as viable therapeutic targets for treating AD.

Herein, we review the function of OX40 and the OX40-OX40L signaling pathway, their role in AD pathogenesis, and emerging therapies targeting OX40-OX40L that may offer insights into the future of AD management.

## 2. Overview of OX40 Signaling Pathways

### 2.1. OX40 Expression

The OX40-OX40L signaling pathway is an instrumental modulator of the adaptive immune response. Antigenic stimulation causes the activation of naïve CD4+ and CD8+ T cells, which induces the transient expression of OX40 [24,25]. OX40 expression principally occurs on activated T cells, and it is not expressed on naïve T cell populations [11]. OX40 expression on activated T cells may be promoted by the production of proinflammatory cytokines that augment the T cell response, including IL-1, IL-2, IL-4, and tumor necrosis factor-alpha (TNF-α) [11]. OX40 expression may also occur on natural killer T (NKT) cells, natural killer (NK) cells, and neutrophils. However, the induction of OX40 expression on these cell types is not well described in the current literature [26].

### 2.2. OX40L Expression

**Figure 1:**
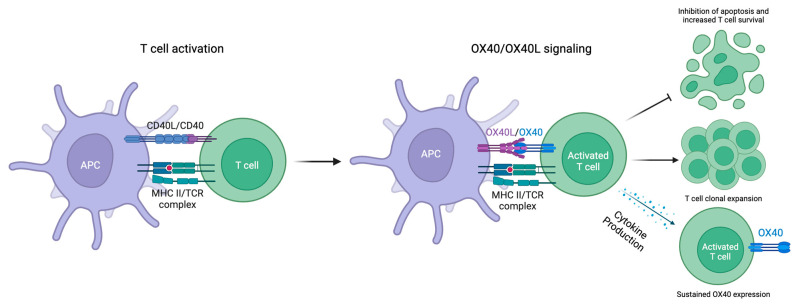
OX40 expression is induced on activated T cells, and OX40L expression is induced on APCs. OX40-OX40L ligation stimulates the clonal expansion of effector CD4+ and CD8+ T cell populations, upregulates the expression of proinflammatory cytokines, and promotes the survival of T cells.

OX40 binds to its endogenous ligand, OX40L, which can be induced on activated B cells, mature conventional dendritic cells (cDCs), plasmacytoid dendritic cells (pDCs), Langerhans cells, and macrophages in response to factors that promote antigen-presenting cell (APC) activation and maturation [27,28,29,30,31]. OX40L expression primarily occurs on professional APCs [11,32,33]. OX40L may also be expressed on type 2 innate lymphoid cells (ILC2s) and non-lymphoid cells, including endothelial cells, fibroblasts, smooth muscle cells, and mast cells [26,34,35,36]. The OX40-OX40L interaction establishes an important costimulatory pathway that induces the clonal expansion of effector CD4+ and CD8+ T cell populations, upregulates the expression of proinflammatory cytokines, and facilitates the generation of memory T cells (Figure 1) [12].

### 2.3. OX40-OX40L Signaling

**Figure 2:**
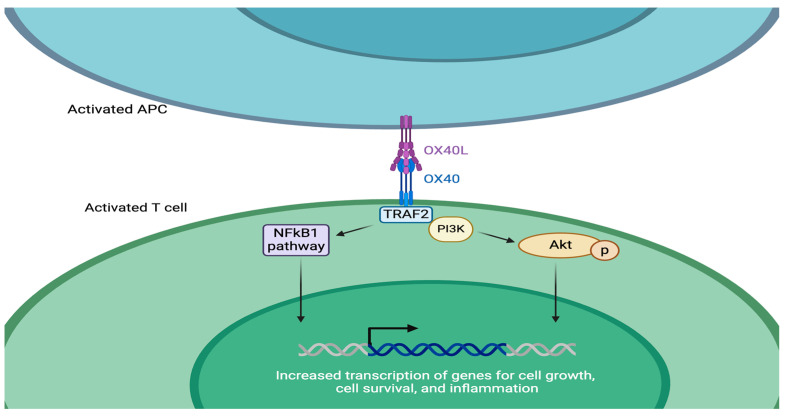
OX40-OX40L ligation activates the PI3K-PKB/Akt and NF-κB1 pathways. These intracellular pathways regulate cellular division and promote cellular growth and T cell survival, which can contribute to inflammation.

As TNFR/TNF superfamily members, both OX40 and OX40L signal through TNF receptor-associated factor (TRAF) adaptor molecules [37]. Specifically, OX40-OX40L ligation generates a signal transduction cascade by activating the phosphoinositide 3-kinase (PI3K) pathway, which results in the downstream phosphorylation and subsequent activation of protein kinase B (PKB, also known as Akt) [38]. The PI3K-PKB/Akt pathway helps regulate cell growth, survival, and metabolism. By controlling PI3K-PKB/Akt activation in T cells, the OX40-OX40L pathway plays an integral role in promoting T cell proliferation, differentiation, and survival (Figure 2). OX40-OX40L signaling also promotes T cell survival through the NF-κB1 pathway, which stimulates intracellular anti-apoptotic protein production [39]. Furthermore, OX40-OX40L signaling contributes to the persistence and reactivation of memory T cell populations [40].

### 2.4. Downstream Effects of OX40-OX40L Signaling

OX40-OX40L ligation also impacts the transcriptional activity of various downstream targets, including forkhead box P3 (FoxP3). The expression of FoxP3 in CD4+ T cells creates a regulatory T cell (Treg) phenotype that suppresses self-reactive T cells in the periphery [41]. Signaling through OX40 has been demonstrated to suppress FoxP3 expression, prevent the induction of new FoxP3+ Tregs from CD4+ T cells, and attenuate the immune suppressor function of existing FoxP3+ CD4+ Tregs [42]. Furthermore, OX40-OX40L signaling has been shown to preferentially act on antigen-specific T cells and promote the primary clonal expansion of these T cell populations [43].

**Figure 3:**
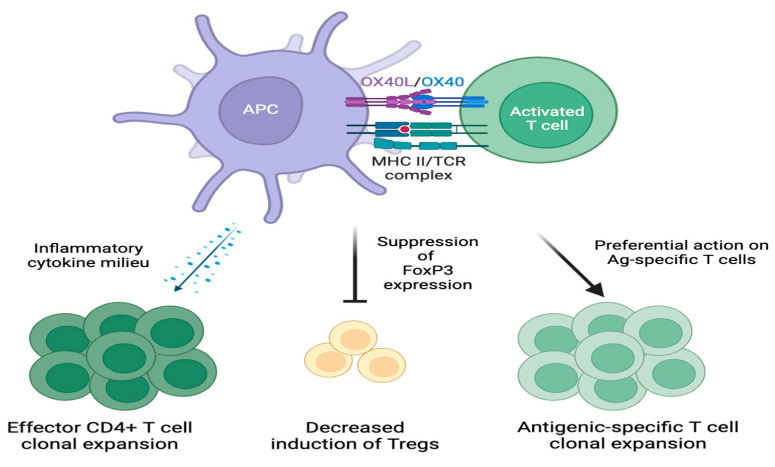
Downstream effects of OX40-OX40L signaling. OX40-OX40L signaling expands effector CD4+ T cell populations when there is a surrounding inflammatory cytokine milieu. OX40-OX40L signaling also decreases the induction of Treg populations through FoxP3 suppression. Additionally, OX40-OX40L signaling promotes the clonal expansion of antigen-specific T cell populations.

In vivo studies have further expanded our understanding of OX40-OX40L signaling in different T cell subsets [44,45]. These studies have demonstrated the impact of the surrounding cytokine milieu on the downstream effects of OX40-OX40L signaling [44,45]. In the presence of cytokines, such as IL-4 and interferon-gamma (IFN-γ), OX40-OX40L signaling leads to the preferential expansion of effector CD4+ T cell populations (Figure 3) [44,45]. In contrast, in the absence of IL-4 and IFN-γ, OX40-OX40L signaling leads to the expansion of the Treg population [44,45]. These studies suggest that in a proinflammatory microenvironment, OX40 will further amplify immune activity due to signaling from inflammatory cytokines. In contrast, in the absence of inflammatory cytokine signaling, OX40 promotes a more immunoregulatory phenotype.

In addition to being a positive effector of T cell growth and survival, OX40-OX40L signaling has also been shown to upregulate the expression of IFN-γ and other proinflammatory cytokine receptors, such as IL-2Rα (CD25), IL-7Rα (CD127), IL-12Rβ2 (CD212), and IL-15Rα (CD215) [37]. These mechanisms describe how the OX40-OX40L axis may amplify the proinflammatory T cell response and highlight its potentially significant role in the pathogenesis of T cell-mediated inflammatory skin diseases, such as AD [46].

## 3. AD Pathogenesis

The OX40-OX40L axis has been implicated in AD pathogenesis [11]. However, many other factors contribute to the complex pathophysiology that characterizes AD [2,47]. The development and progression of AD may represent an interplay between genetic predisposition, environmental exposure, epidermal barrier dysfunction, microbial dysbiosis, and immune system dysregulation [1,4,48,49].

### 3.1. Phenotypic Variance

The multifactorial etiology behind AD development lends itself to a heterogeneous clinical course and presentation [8,50,51]. Several characteristics may contribute to the wide range of observed AD phenotypes, including race, ethnicity, age at disease onset, disease stage (i.e., duration), geographic location, and IgE levels [4,9]. An analysis of the underlying molecular endotype that drives the clinical presentation of AD may help explain its phenotypic variance in different populations [52]. For example, preliminary work comparing AD patients with different racial backgrounds has shown that Asian AD patients tend to demonstrate greater T helper 17 (T_H_17) axis activation than African American and European American AD patients [9,52]. However, the results of these studies need to be validated with large-scale, longitudinal research [53]. Age may also influence molecular endotype as older AD patients have been shown to exhibit decreased activation of the T_H_2 and T_H_22 axes compared to younger AD patients [54]. These findings emphasize the complexity of AD and highlight potential factors that can contribute to its pathogenesis.

### 3.2. Skin Barrier Dysfunction

Patients with AD may experience skin barrier dysfunction due to several reasons, including structural protein deficiency, imbalanced stratum corneum enzyme activity, abnormal lipid composition and organization, and defective tight junction proteins [1,2,55,56]. A defective epidermal barrier can lead to transepidermal water loss that may dehydrate the skin and increase its susceptibility to penetration by irritants and allergens [2,9,56,57]. A compromised epidermal barrier may also impact the microbiome of organisms that constitute our natural skin flora [49].

### 3.3. Skin Dysbiosis

The skin is inhabited by numerous commensal microbes that help regulate its homeostatic functions, such as generating immune responses and preventing the overgrowth of virulent pathogens [49,58]. *Staphylococcus*, *Corynebacterium*, and *Propionibacterium* comprise around 60% of the bacterial species in healthy skin flora, whereas *Malassezia* species represent the most abundant fungi [59]. Compared to the skin microbiome of healthy patients, the bacterial composition of the skin microbiome in AD patients is altered [59]. Epidermal barrier impairment facilitates microbiota disruption, triggering immunologic processes that may contribute to AD pathogenesis [49]. Cutaneous dysbiosis in AD is characterized by decreased microbial diversity and greater proliferation of *Staphylococcal* species, particularly *Staphylococcus aureus* (*S. aureus*) [9,49,60].

Several studies have shown that the extent of *S. aureus* colonization positively correlates with AD disease severity [49,58,59,61]. This relationship may be due to the role that *S. aureus* plays in AD pathogenesis. For example, *S. aureus* stimulates endogenous protease activity and expression in keratinocytes [59,62]. Increased proteolysis in keratinocytes facilitates filaggrin cleavage, which decreases epidermal barrier integrity and increases its vulnerability to penetration [59,62,63].

Additionally, *S. aureus* produces pathogenic virulence factors, including alpha toxin (α-toxin/α-hemolysin/H1a) and delta toxin (δ-toxin/δ-hemolysin) [49,58]. α-Toxin forms pores in the cellular membrane of various types of cells, including epithelial cells, endothelial cells, and neutrophils [64]. Pore formation in the epidermal barrier stimulates the secretion of proinflammatory cytokines that generate an immune response [58,64]. δ-toxin induces mast cell degranulation and IgE production, which promotes T_H_2 signaling and T_H_2-mediated cytokine production [49,58,65]. Therefore, dysbiosis of the cutaneous microbiome may facilitate AD pathogenesis by exacerbating skin barrier dysfunction and potentiating inflammation.

### 3.4. Immune System Dysregulation

The disruption of the epithelial barrier stimulates toll-like receptors (TLRs) expressed on keratinocytes and APCs in the skin, which subsequently activates the innate immune system [1,56]. The activation of the innate immune system leads to the release of antimicrobial peptides, proteases, extracellular matrix proteins, and alarmins such as IL-1α, IL-25, IL-33, macrophage-derived chemokine (MDC), thymus and activation-regulated chemokine (TARC), and thymic stromal lymphopoietin (TSLP) [1,56,66]. These molecules activate dendritic cells, Langerhans cells, and ILC2s, which stimulate T_H_2 cells [1].

The overactivation of the T_H_2 cell response increases the secretion of several cytokines that characterize early-stage AD, including IL-4, IL-5, and IL-13 [3]. These cytokines disrupt the epidermal barrier by decreasing the expression of molecules that promote normal barrier function (e.g., structural proteins, lipid synthetic enzymes, and antimicrobial peptides) and help stimulate IgE class-switching and eosinophil production [1,55,56,57,67]. T_H_2 cells also induce the secretion of IL-31, a cytokine that mediates pruritus, which exacerbates skin inflammation through repetitive scratching that induces trauma and triggers additional cytokine release (i.e., the “itch-scratch” cycle) [8,22,57,68].

AD inflammation is primarily mediated by T_H_2 cell-driven immune pathways; however, other T helper cell populations (e.g., T_H_1, T_H_17, and T_H_22 cells) also contribute to disease pathogenesis and chronicity [11,69]. The upregulation of T_H_2 and T_H_22 cells characterizes the acute phase of inflammation. In contrast, the chronic phase of inflammation is marked by the upregulation of T_H_1, T_H_2, T_H_17, and T_H_22 cells [3,11,69]. These distinct T helper cell environments exemplify the heterogeneity of immune dysregulation that drives AD pathogenesis. Therefore, molecules like OX40 that can stimulate the proliferation of multiple subsets of T helper cells may play a significant role in the pathogenesis and progression of AD [11,15].

## 4. OX40-OX40L Signaling in Atopic Dermatitis

Skin barrier disruption is a hallmark of AD that leads to the release of epithelial cell-derived cytokines from keratinocytes found in the epidermis, including TSLP, IL-25, and IL-33 [11,57,70,71]. These cytokines are overexpressed in the skin of patients with AD, likely due to the effects of multiple factors, including epigenetic modifications (e.g., the hypomethylation of TSLP promoters) and exposure to environmental allergens [57,72].

### 4.1. T Helper 2 Cells

OX40 and OX40L form a key immunopathogenic signaling pathway that may influence the development of AD through numerous cellular processes [9,11]. Skin barrier dysfunction is a key first step in activating CD4+ T cells and, subsequently, the OX40-OX40L signaling pathway. The disruption of the skin barrier allows for the entry of foreign antigens through the skin which prime T_H_2 cells [11]. In addition, damage to epithelial cells induces the production of proinflammatory cytokines and chemokines, including thymic stromal lymphopoietin (TSLP), IL-25, and IL-33 [11,15]. Among them, TSLP primarily induces OX40L expression on APCs; OX40L-expressing APCs facilitate the activation of CD4+ T cells and OX40 expression on T cells [11,15]. IL-25 and IL-33 produce cytokines that drive type 2 inflammation and further upregulate OX40L expression [71]. The ligation of OX40L to OX40 on activated CD4+ T cells drives the proliferation of effector T helper cell populations, principally T_H_2 cells, that produce cytokines implicated in early AD pathogenesis, such as IL-4 and IL-13 [1,12,73].

IL-4 and IL-13 are proinflammatory cytokines secreted by T_H_2 cells that downregulate filaggrin, loricrin, and involucrin production in the epidermis [9,74]. The downregulation of these structural proteins compromises skin barrier integrity [23,33,57]. The subsequent disturbance of the epidermal barrier establishes a feedback loop that increases TSLP production and stimulates OX40L expression, thus contributing to the T_H_2 cell predominance that characterizes early-stage AD [3].

### 4.2. T Helper 1, T Helper 17, and T Helper 22 Cells

OX40 expression in other activated T helper cell populations, including T_H_1, T_H_17, and T_H_22 cells, may help facilitate the transition from an acute to chronic inflammatory state [15]. Maximal OX40 expression typically occurs within 1-4 days after antigenic stimulation [12,75]. This temporal relationship allows OX40 to contribute to both the acute (i.e., days 1-3) and chronic (i.e., after 72 h) phases of AD inflammation [3,11].

During the acute phase of inflammation, OX40-OX40L signaling promotes T_H_2 cell differentiation [3,11]. Activated T_H_2 cells express OX40 and secrete cytokines that further disrupt the epidermal barrier, which activates APCs that subsequently express OX40L [3,11,12,15,71,76]. OX40-OX40L ligation facilitates the proliferation of additional T_H_2 cells that help maintain inflammation; prolonged inflammation sustains OX40 and OX40L expression on T helper cells and APCs [3,11,15].

**Figure 4:**
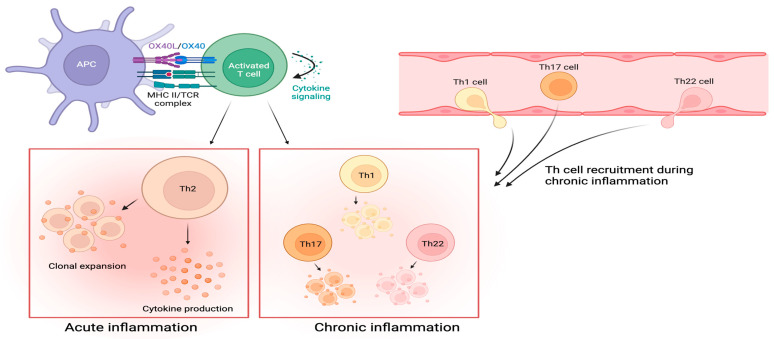
Two phases of inflammation characterize AD. During the acute phase, OX40-OX40L ligation on activated T cells facilitates T_H_2-predominant signaling and differentiation, which produces type 2 inflammatory cytokines. The shift to the chronic phase is characterized by the recruitment of T_H_1, T_H_17, and T_H_22 cells expressing OX40. OX40-OX40L ligation on these activated T helper cell populations leads to effector cell proliferation and cytokine production that maintain the inflammatory response.

During the chronic phase of inflammation, other T helper cell subsets that express OX40 (i.e., T_H_1, T_H_17, and T_H_22 cells) are recruited to the inflammatory microenvironment [3]. Continued OX40-OX40L signaling allows for the proliferation of effector T_H_1, T_H_17, and T_H_22 cell populations (Figure 4) [3,11]. Effector T_H_1, T_H_17, and T_H_22 cells upregulate the production of IFN-γ, IL-17, and IL-22. These cytokines help mediate the progression of acute to chronic AD through keratinocyte proliferation, epidermal thickening, and the production of mediators that recruit T cells into the skin [1,46,52,69,71,77].

### 4.3. Memory T Cells

In addition to promoting T cell differentiation and proliferation, the OX40-OX40L pathway also contributes to the generation of memory T cells that may further modulate AD pathogenesis. Many effector T cells undergo apoptosis following a primary immune response; however, OX40-OX40L signaling mediates the transition of some effector T cells into resting memory T cells [11,12,15]. Although the exact mechanism is not fully understood, the anti-apoptotic effects of OX40-OX40L signaling likely contribute to the enhanced survival of memory T cells after the initial response to antigen presentation [11,12,20].

**Figure 5:**
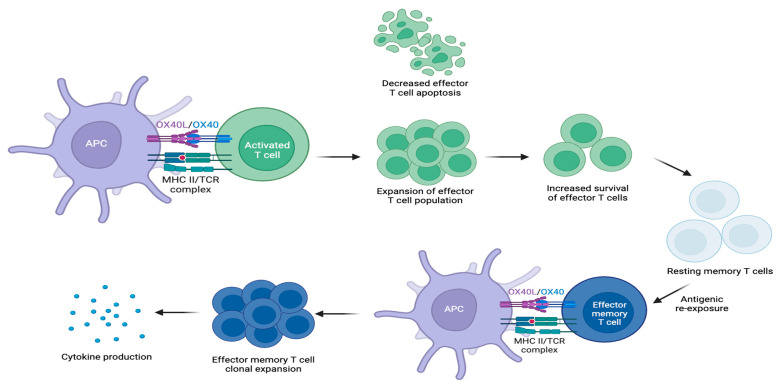
OX40-OX40L signaling causes effector T cell proliferation, which generates a primary immune response. Effector T cells undergo apoptosis following a primary immune response; however, OX40-OX40L signaling mediates the transition of some into resting memory T cells. Re-exposure to the same antigen converts resting memory T cells into effector memory T cells expressing OX40. OX40-OX40L ligation on effector memory T cells causes effector memory T cell proliferation, leading to a secondary immune response.

Re-exposure to the same antigen activates resting memory T cells and converts them into effector memory T cells that rapidly express OX40 [12,15,78]. Ligation with OX40L facilitates the expansion of effector T helper cell populations that secrete new proinflammatory cytokines (Figure 5) [15]. Effector memory T cells expressing OX40 are upregulated in AD lesions, which may offer a potential immunologic explanation for disease recurrence (i.e., flares) [15,79,80]. These findings demonstrate how OX40 may facilitate the chronicity of AD through the maintenance of a robust memory T cell population [11].

### 4.4. Suppression of Apoptosis

The OX40-OX40L axis may also contribute to the pathogenesis of AD through the suppression of T cell apoptosis. Under normal physiologic conditions, apoptosis prevents sustained inflammation through tightly regulated processes influenced by different signal transduction cascades and molecules [81,82,83,84,85]. T cell apoptosis is dysregulated in AD, which facilitates prolonged inflammation and disease progression [86,87].

OX40 induces the sustained activation of intracellular anti-apoptotic pathways in T cells. The nuclear factor-κB (specifically, NF-κB1) and PI3K-PKB/Akt pathways are key modulators of cell division and apoptosis that respond to OX40 signaling [12]. The NF-κB signal transduction cascade promotes inflammation through two distinct pathways: the canonical (NF-κB1) pathway and the non-canonical/alternative pathway [81,88]. The OX40-mediated stimulation of the NF-κB1 pathway increases the production of anti-apoptotic proteins (e.g., survivin, Bcl-2, and Bcl-xL) that regulate cell division and prolong T cell survival [39]. The OX40-mediated activation of the PI3K-PKB/Akt pathway also upregulates the expression of these anti-apoptotic factors [39,89]. The activation of these pathways may lead to cellular survival and subsequent memory T cell accumulation after an initial effector T cell response [12,24,32,90].

### 4.5. Modulation of OX40-OX40L Signaling

Collectively, these mechanisms suggest a viable pathophysiological link between OX40-OX40L and AD. The OX40-OX40L axis modulates processes integral to AD pathogenesis that promote T cell differentiation and proliferation and the survival of multiple subsets of T helper cells [12]. Disrupting the costimulatory interaction between OX40 and OX40L may lead to a diminished T cell response [32]. As such, the inhibition of OX40-OX40L ligation is a potentially significant area of therapeutic interest for the management of AD.

## 5. Therapeutic Targets and Emerging Therapies

The treatment landscape for moderate-to-severe AD has greatly expanded due to the development of systemic therapies that inhibit specific cytokines or small molecules implicated in AD pathogenesis. A few examples of these novel treatments include biologics that inhibit IL-4Rα (dupilumab) and IL-13 (tralokinumab), as well as JAK inhibitors [1,8,9,11,91]. Despite this recent drug discovery, a therapeutic need exists for patients with moderate-to-severe AD who do not adequately respond to available therapies [11,91].

**Figure 6:**
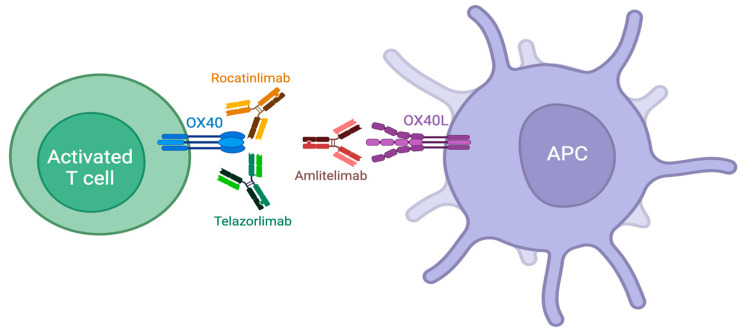
Novel therapies targeting the interaction between OX40 and OX40L include monoclonal antibodies against OX40 (rocatinlimab and telazorlimab) and OX40L (amlitelimab).

Several emerging AD therapies targeting the OX40-OX40L pathway are currently in development, including the monoclonal antibodies rocatinlimab (AMG 451/KHK4083), telazorlimab (ISB 830/GBR 830), and amlitelimab (KY1005/SAR445229) (Figure 6).

### 5.1. Rocatinlimab

Rocatinlimab is a fully human anti-OX40 monoclonal antibody that selectively depletes OX40+ activated T cells, downregulating T_H_1-, T_H_2-, T_H_17-, and T_H_22-mediated inflammation [91]. In a phase 2b trial, rocatinlimab demonstrated good efficacy and safety profiles (NCT03703102) [23]. This multi-center, double-blind, placebo-controlled trial included 274 adult subjects with moderate-to-severe AD and an inadequate response to topical treatments. The primary endpoint was the least-squares mean percent change in the Eczema Area and Severity Index (EASI) score. The EASI scores ranged from 0 to 72, with higher scores representing increased severity from baseline to week 16 between the placebo and treatment groups. Subjects in this trial were randomized (1:1:1:1:1) to receive rocatinlimab in doses of (1) 150 mg subcutaneously every four weeks (*n* = 52), (2) 600 mg subcutaneously every four weeks (*n* = 52), (3) 300 mg subcutaneously every two weeks (*n* = 52), or (4) 600 mg subcutaneously every two weeks (*n* = 54) or (5) a placebo for 18 weeks (*n* = 57). The treatment period was followed by an 18-week active-treatment extension and a subsequent 20-week safety follow-up period.

At week 16, all treatment groups demonstrated significant improvement in their EASI scores relative to the placebo group. When compared to the placebo group’s mean decrease in the EASI score of −15.0% (95% CI, −28.6%–−1.4%), (1) the group receiving 150 mg subcutaneously every four weeks demonstrated a significantly higher decrease in the EASI score of −48.3% (95% CI, −62.2%–−34.0%; *p* = 0.0003), (2) the group receiving 600 mg subcutaneously every four weeks demonstrated a significantly higher decrease in the EASI score of −49.7% (95% CI, −64.3%–−35.2%; *p* = 0.0002), (3) the group receiving 300 mg subcutaneously every two weeks demonstrated a significantly higher decrease in the EASI score of −61.1% (95% CI, −75.2%–−47.0%; *p* < 0.0001), and (4) the group receiving 600 mg subcutaneously every two weeks demonstrated a significantly higher decrease in the EASI score of −57.4% (95% CI, −71.3%–−43.4%; *p* < 0.0001). Treatment-emergent adverse events were mild, and no deaths were reported. The most common adverse events reported in ≥5% of the rocatinlimab-treated groups included pyrexia, chills, headache, aphthous ulcer, and nausea [23].

Rocatinlimab is currently undergoing placebo-controlled, double-blind trials and open-label phase 3 trials in adults and adolescents with moderate-to-severe AD to continue to assess the efficacy, safety, and tolerability of this new intervention (NCT05633355, NCT05651711, NCT05398445, NCT05899816, NCT05724199, NCT05882877, NCT05704738).

### 5.2. Telazorlimab

Telazorlimab is a humanized anti-OX40 monoclonal antibody that may selectively deplete activated T cells to interrupt the inflammatory pathways associated with AD [92,93]. In a phase 2b trial, telazorlimab demonstrated good efficacy and safety profiles (NCT03568162) [92]. This multi-center, double-blind, placebo-controlled trial included adult subjects with moderate-to-severe AD and consisted of two parts. The primary endpoint was the least-squares mean percent change in the EASI score from baseline to week 16 between the placebo and treatment groups.

Part 1 included 313 subjects who were randomized (1:1:1:1) to receive telazorlimab subcutaneously (1) with a loading dose of 600 mg and then 300 mg every two weeks (*n* = 76), (2) with a loading dose of 600 mg and then 300 mg every four weeks (*n* = 78), or (3) with a loading dose of 150 mg and then 75 mg every four weeks (*n* = 77) or (4) a placebo (*n* = 80) for 16 weeks. The treatment period was followed by a 38-week open-label extension in which all participants received telazorlimab 300 mg subcutaneously every two weeks.

Part 2 included 149 subjects who were randomized (1:1) to receive telazorlimab subcutaneously with a loading dose of 1200 mg and then 600 mg every two weeks (*n* = 75) or a placebo (*n* = 74) for 16 weeks. This treatment period was followed by a 38-week open-label extension in which all participants received telazorlimab 600 mg subcutaneously every two weeks. All subjects from parts 1 and 2 were monitored afterward for a 12-week safety follow-up period.

At 16 weeks, the EASI scores were significantly improved in the two high-dose treatment groups compared to the placebo group. In part 1, the high-dose group receiving 300 mg every two weeks demonstrated a significantly higher decrease in the mean EASI score of −54.4% (SE 5.1; *p* = 0.008) in comparison to the placebo group’s mean decrease in the EASI score of −34.2% (SE 5.5). In part 2, the high-dose group receiving 600 mg every two weeks demonstrated a significantly higher decrease in the mean EASI score of −59.0% (SE 4.6; *p* = 0.008) in comparison to the placebo group’s mean decrease in the EASI score of −41.8% (SE 4.7).

Among the low-dose treatment groups, no significant differences were found in EASI scores between the group receiving 300 mg every four weeks and the placebo group (−48.6% [SE 5.4] versus −34.2% [SE 5.7]; *p* = 0.061) or the group receiving 75 mg every four weeks and the placebo group (−31.0% [SE 5.7] versus −34.2% [SE 5.7]; *p* = 0.691). The adverse event rates appeared to be distributed similarly between all treatment and placebo arms. Across parts 1 and 2 of this study, the most common treatment-emergent adverse events, which were defined as occurring in >5% of the telazorlimab-treated groups, were AD, nasopharyngitis, upper respiratory tract infection, headache, pruritus, and fatigue [92]. Similar rates of AD exacerbation, nasopharyngitis, upper respiratory tract infection, headache, and urinary tract infection also occurred in the placebo groups [92].

There are currently no ongoing phase 3 trials that are further investigating the utility of telazorlimab in the management of AD.

### 5.3. Amlitelimab

Amlitelimab is a fully human anti-OX40L monoclonal antibody that binds to OX40L. Amlitelimab binding to OX40L blocks its interaction with OX40, which inhibits the activation of T cell-mediated inflammation. Amlitelimab also reduces the proinflammatory activity of APCs by blocking the OX40L back signaling pathway [91].

In a phase 2a trial, amlitelimab demonstrated good efficacy and safety profiles (NCT03754309) [94]. This multi-center, double-blind, placebo-controlled trial included 89 adult subjects with moderate-to-severe AD. The primary endpoint was the least-squares mean percent change in the EASI score from baseline to week 16 between placebo and treatment groups. The secondary endpoints included changes in serum IL-22 levels from baseline to week 16 between the treatment and placebo groups. Subjects were randomized (1:1:1) to receive amlitelimab subcutaneously (1) with a 200 mg loading dose and 100 mg every four weeks (*n* = 27), (2) with a 500 mg loading dose and 250 mg every four weeks (*n* = 27), or (3) a placebo (*n* = 24) for 12 weeks. The treatment period was followed by a 24-week safety follow-up period.

At week 16, the low-dose treatment group (100 mg every four weeks) demonstrated a significant decrease in the mean EASI score of −80.12% (95% CI, −95.55%–−54.60%; *p* = 0.009) compared to the placebo group’s mean decrease in the EASI score of −49.37% (95% CI, −66.02%–−32.72%). However, at week 16, the high-dose treatment group (250 mg every four weeks) demonstrated a non-significant decrease in the mean EASI score of −69.97% (95% CI, −85.04%–−54.60%; *p* = 0.07) as compared to the placebo group’s mean decrease in the EASI score of −49.37% (95% CI, −66.02%–−32.72%). Additionally, serum IL-22 levels at week 16 were found to be significantly reduced from baseline in the low-dose treatment group (*p* < 0.001) and high-dose treatment group (*p* < 0.001). No reduction in serum IL-22 levels was found for the placebo-treated group between baseline and week 16. The safety profile was unremarkable. The most common adverse events among the amlitelimab-treated groups, occurring with ≥5% frequency compared to the placebo group, were mild and included headache, hyperhidrosis, upper respiratory tract infection, pyrexia, increased aspartate aminotransferase, and iron deficiency anemia [94].

The preliminary results of the phase 2b trial of amlitelimab were presented at the 2023 European Academy of Dermatology and Venereology (EADV) Congress (NCT05131477) [95]. This multi-center, double-blind, placebo-controlled trial included 390 subjects with moderate-to-severe AD. In this dose-ranging study, subjects were randomized (1:1:1:1:1) to receive amlitelimab subcutaneously as follows: (1) 250 mg every four weeks with a 500 mg loading dose (*n* = 77), (2) 250 mg every four weeks without a loading dose (*n* = 78), (3) 125 mg every four weeks without a loading dose (*n* = 77), (4) 62.5 mg every four weeks without a loading dose (*n* = 79), or (5) a placebo (*n* = 79). The primary endpoint was the least-squares mean percent change in the EASI score from baseline to week 16 between the placebo and treatment groups [95].

At week 16, all treatment groups demonstrated significant improvement in the EASI score relative to the placebo group. Compared to the placebo group, the decrease in the least-squares mean percent change in the EASI score was −32.1% (95% CI, −43.9%–−20.3%; *p* < 0.0001) for the group receiving 250 mg every four weeks with a 500 mg loading dose, −27.3% (95% CI, −39.1%–−15.6%; *p* < 0.0001) for the group receiving 250 mg every four weeks without a loading dose, −22.2% (95% CI, −34.0%–−10.4%; *p* = 0.0002) for the group receiving 125 mg every four weeks without a loading dose, and −30.2% (95% CI, −41.9%–−18.5%; *p* < 0.0001) for the group receiving 62.5 mg every four weeks without a loading dose. Amlitelimab was well tolerated in all the treatment groups [95].

Amlitelimab is currently undergoing placebo-controlled, double-blind phase 3 trials in adults with moderate-to-severe AD (NCT06181435, NCT06130566).

Table 1 summarizes the phase 2b clinical trials studying the effects of OX40-OX40L pathway inhibition in adults with moderate-to-severe AD.

## 6. Conclusions

In conclusion, the OX40-OX40L axis appears to strongly influence immune pathways contributing to skin inflammation in AD. Numerous cellular mechanisms support the significant role of OX40 and OX40L in AD pathogenesis. The OX40-OX40L pathway allows multiple classes of T helper cells to proliferate. These T helper cell subsets secrete cytokines that impair epidermal barrier function, dysregulate the immune response, and maintain inflammation. OX40-OX40L ligation also promotes the formation of memory T cells. Once activated, these T cells can further augment the production of proinflammatory cytokines. In addition, the OX40-OX40L pathway sustains the activation of anti-apoptotic intracellular pathways, allowing more T cells to potentially contribute to disease progression and chronicity.

The heterogeneity of AD pathogenesis may limit the effectiveness of available treatments, and options are sparse for patients with moderate-to-severe AD who do not respond to existing therapies. Disrupting the OX40-OX40L interaction may attenuate the T cell response and offer therapeutic utility for AD patients. AD pathogenesis is heterogeneous among different patient populations. While traditionally thought of as a T_H_2-mediated disease, AD in different populations may be differentially dominated by other pathways such as T_H_1, T_H_17, and T_H_22 [9,52,54]. OX40-OX40L signaling augments the expansion and survival of several T helper cell subsets [91], therefore sustaining the proinflammatory state across different phenotypes of AD patients.

Currently available systemic therapies for AD are mostly limited to targeting T_H_2 cell-driven inflammation. In contrast, therapies targeting OX40-OX40L signaling may provide additional therapeutic benefits to patients with varying AD phenotypes and endotypes, including those who do not adequately respond to treatments that target specific components of the T_H_2 pathway.

Anti-OX40-OX40L therapies target AD pathogenesis more upstream by limiting the proliferation of T_H_1, T_H_2, T_H_17, and T_H_22 cells [91]. As such, anti-OX40-OX40L therapies may provide long-term durability over current AD treatments and may be administered infrequently while maintaining efficacy [23].

Lastly, regarding differentiating OX40-OX40L inhibitors from existing AD biologics in terms of safety profiles, the OX40-OX40L inhibitors do not have the adverse effect of conjunctivitis, which is related to the use of IL-4 and/or IL-13 inhibitors [96,97]. Of note, it is important to consider that the safety profile among the OX40-OX40L inhibitors differs slightly, and this may be related to whether the inhibitor depletes T cell populations. However, in the absence of larger phase 3 studies at this time, it is difficult to fully appreciate safety differences among the OX40-OX40L inhibitors.

Several clinical trials are studying novel treatments targeting OX40 and OX40L in AD patients. While these treatments have demonstrated encouraging results in early-phase trials, continued research is needed to assess their long-term safety, tolerability, and efficacy in patients with moderate-to-severe AD. These promising therapies may significantly impact the future of AD management and enhance disease control for more AD patients.

## Figures and Tables

**Table 1:** Phase 2b clinical trials of emerging therapies targeting the OX40-OX40L pathway.

Therapeutic Agent	Site of Action	Study Design		Primary Endpoint	Study Arms	Results
Rocatinlimab	OX40	NCT03703102 [23] Phase 2b multi-center, double-blind, placebo-controlled trial of 274 adults with moderate-to-severe AD randomized 1:1:1:1:1		Least-squares mean percent change in EASI from baseline to week 16	Placebo	−15.0% (95%CI, −28.6%–−1.4%)
					No LD 150 mg q4weeks	−48.3% (95%CI, −62.2%–−34.0%) *p* = 0.0003
					No LD 600 mg q4weeks	−49.7% (95%CI, −64.3%–−35.2%) *p* = 0.0002
					No LD 300 mg q2weeks	−61.1% (95%CI, −75.2%–−47.0%) *p* < 0.0001
					No LD 600 mg q2weeks	−57.4% (95%CI, −71.3%–−43.4%) *p* < 0.0001
Telazorlimab	OX40	NCT03568162 [93] Phase 2b multi-center, double-blind, placebo-controlled trial of adults with moderate-to-severe AD	Part 1 313 subjects randomized 1:1:1:1	Least-squares mean percent change in EASI from baseline to week 16	Placebo	−34.2% (SE 5.5)
					LD: 150 mg 75 mg q4weeks	−31.0% (SE 5.7) *p* = 0.691
					LD: 600 mg 300 mg q4weeks	−48.6% (SE 5.4) *p* = 0.061
					LD: 600 mg 300 mg q2weeks	−54.4% (SE 5.1) *p* = 0.008
			Part 2 149 subjects randomized 1:1	Least-squares mean percent change in EASI from baseline to week 16	Placebo	−41.8% (SE 4.7)
					LD: 1200 mg 600 mg q2weeks	−59.0% (SE 4.6) *p* = 0.008
Amlitelimab	OX40L	NCT05131477 [95] Phase 2b multi-center, double-blind, placebo-controlled trial of 390 adults with moderate-to-severe AD randomized 1:1:1:1:1		Least-squares mean percent change in EASI from baseline to week 16	Placebo	−29.4%
					LD: 500 mg 250 mg q4weeks	−61.5% *p* < 0.0001
					No LD 250 mg q4weeks	−56.8% *p* < 0.0001
					No LD 125 mg q4weeks	−51.6% *p* = 0.0002
					No LD 62.5 mg q4weeks	−59.6% *p* < 0.0001

Abbreviations: AD, atopic dermatitis; EASI, Eczema Area and Severity Index; LD, loading dose; q2weeks, every two weeks; q4weeks, every four weeks; 95%CI, 95% confidence interval; SE, standard error.

## Data Availability

Not applicable.
